# Virtual focus groups among individuals with use disorders: assessing feasibility and acceptability in an underserved clinical population

**DOI:** 10.3389/fpsyt.2024.1352300

**Published:** 2024-03-11

**Authors:** Cecilia L. Bergeria, Brandon Park, Prem Umang Satyavolu, Kelly E. Dunn, Robert H. Dworkin, Eric C. Strain

**Affiliations:** ^1^Department of Psychiatry and Behavioral Sciences, Johns Hopkins University School of Medicine, Baltimore, MD, United States; ^2^Department of Anesthesiology and Perioperative Medicine, University of Rochester, Rochester, NY, United States

**Keywords:** focus groups, virtual, stimulant use disorder, opioid use disorder, remote data collection

## Abstract

**Objective:**

There are substantial barriers to conducting research among individuals with stigmatized and complicated health conditions like substance use disorders. These barriers slow progress when developing, refining, and assessing interventions to better treat underserved populations. Virtual focus groups are an innovative method for collecting data from individuals via a discreet and accessible platform which can inform novel as well as existing treatment approaches. This article reports on the feasibility and acceptability of virtual focus groups as a mechanism to recruit and engage geographically and demographically diverse samples of participants with substance use disorders that are otherwise logistically difficult to assess.

**Method:**

Participants were assessed for eligibility for a virtual focus group study based on demographic features, drug use history, and psychiatric history via a remote, interview-based screening. Focus groups were completed anonymously without video or name-sharing. Discussion contributions, quantified with number of times speaking and total number of words spoken, were compared across gender, and treatment status. Participants provided quantitative and qualitative feedback on the focus group experience in a follow-up survey.

**Results:**

Focus groups (*N*=26) based in geographical areas throughout the United States were conducted with 88 individuals with opioid use disorder or stimulant use disorder. Discussion contributions were comparable between genders and among individuals in treatment versus those seeking treatment. A follow-up survey (*n*=50, 57% of focus group participants) reflected high levels of enjoyment, comfort, and honesty during focus group discussions.

**Discussion:**

Findings suggest virtual focus groups can be an effective and efficient tool for substance use research.

## Introduction

Research is sorely needed to improve and develop treatments for individuals with substance use disorders (SUDs). Outstanding treatment needs are especially evident in the context of escalating morbidity and mortality associated with the worsening opioid epidemic and a corresponding rise in stimulant use disorder (1). However, there are substantial barriers to conducting research among individuals with opioid use disorder (OUD) and stimulant use disorder (StUD) which slows progress on developing innovative interventions.

Even before the SARS-CoV-2 pandemic, there were significant difficulties associated with conducting research among individuals with SUDs. For instance, individuals with SUD experience a considerable amount of stigma, which has been found to deter individuals from interacting with mental health or medical professionals and/or from openly discussing their substance use problems (2). Individuals with SUDs are also deterred from easily participating in research because of social and structural barriers or competing demands (e.g., unstable housing, unemployment, lack of transportation, childcare needs) (3). Together these compounding barriers indicate a need to develop unique accommodations and considerations for persons with SUD seeking to enroll into research studies. Research on SUDs has also been limited by inadequate and non-representative sampling. For example, several systematic reviews have determined that participants in randomized clinical trials for the treatment of SUDs were not representative of the United States (U.S.) and were mostly white and male (4–6). This suggests treatments shown to be effective in these trials benefit individuals who are white and male, but that the efficacy for other demographic groups remains undetermined.

The SARS-CoV-2 (e.g., COVID-19) pandemic has accelerated the development and utilization of virtual approaches in both clinical treatment and research settings. Conducting research remotely, when possible and appropriate, could mitigate the challenges associated with participant enrollment and representative sampling. First, remote appointments eliminate the need for reliable transportation and reduces the time commitment for participating in research (7). Completing remote appointments also allows participants to participate from home, potentially removing the need for arranging childcare, which disproportionately impacts women (8, 9). Further, conducting research remotely allows for recruitment from geographic locations other than the research team’s immediate area, which can constrain the ability to enroll a diverse sample (7).

To date, the majority of research on substance use disorders that utilize virtual focus groups enrolls providers or professionals who work with individuals with substance use disorders (10–12). It is unclear whether recruitment and retention for this research method is or is not particularly challenging with persons who are actively using substances, as access to the Internet is not always stable and their comfort with video conferencing and remote survey completion can be variable (13, 14).

We collected acceptability and feasibility outcomes for conducting remote focus groups among persons with SUDs. The purpose of the present focus groups was to collect qualitative data as a necessary part of the development process required by the Food and Drug Administration (FDA) to create clinical outcome assessments (COAs), in this case for a measure of craving. Within the FDA’s framework, there is an emphasis on the collection of patient-centered feedback to guide the development of valid and reliable measures to address public health needs (15–17). Therefore, to collect qualitative data among a diverse and representative sample, we recruited individuals with opioid use disorder (OUD) and stimulant use disorder (StUD) throughout different regions in the United States to participate in focus group discussions on craving using remote methodology. Demographics were collected to determine the ability of this method to recruit more diverse samples of participants. Given the novelty of this approach, the following report outlines (1) descriptions of the method for recruiting, screening, and conducting remote focus groups and (2) data on the feasibility and acceptability of virtual focus groups among individuals with OUD and StUD.

## Method

### Participants

Participants were individuals with moderate to severe OUD and/or StUD based on *Diagnostic and Statistical Manual of Mental Disorders, Fifth Edition* (*DSM-5*) criteria, and who indicated they were treatment seeking or receiving treatment for opioid or stimulant use (18). Eligible participants were able to understand and speak English and willing and able to participate in a remote focus group using Zoom video teleconferencing. Participants were recruited from rural, suburban rand urban settings located in the following areas across the United States: Atlanta, GA, Baltimore, MD, Boston, MA, Columbus, OH, Concord, NH, Denver, CO, Detroit, MI, Minneapolis, MN, New Orleans, LA, Panama City, FL, Phoenix, AZ, Seattle, WA, San Francisco, CA, and St. Louis, MO.

Regarding sample size, a series of independent, sequential focus groups were conducted until thematic saturation was achieved across subgroups. Thematic saturation was assessed after completion of a predetermined number of focus groups per subgroup (19). Primary outcomes are not summarized here as this paper focuses on the method, its feasibility, and its acceptability. Data included in this manuscript can be made available by request by contacting the corresponding author. This study was not preregistered.

### Recruitment

Participants were recruited using online ads (e.g., Craigslist, Facebook, Google ads) targeting specific geographical areas of interest. Single focus groups were restricted to individuals from the targeted geographical areas. Interested participants contacted study staff via phone and completed a pre-screening interview or completed the online pre-screening survey accessible through a link included with online advertisements. Pre-screening took no more than 5 minutes and assessed whether individuals were (1) 18 years or older, (2) currently self-described as having an OUD or StUD, (3) currently in treatment or seeking treatment for OUD or StUD, and (4) not currently pregnant. Participants eligible after pre-screening were scheduled for a 1-hour screening appointment to complete informed consent and determine final eligibility.

### Consenting and eligibility screening

Consent and screening were conducted via phone or videoconferencing (e.g., Zoom), based on participant preference. Before screening interviews, trained research team members read an Institutional Review Board (IRB)-approved oral consent script aloud for the participant and asked throughout and at the end whether the participant had any questions or needed any clarification. Waiver of consent documentation (i.e., signed informed consent) and the usage of oral consent was approved by Johns Hopkins IRB because the study was determined to pose no more than minimal risk to the participant and was to involve no procedure for which written consent is normally required outside the research context.

To decrease the likelihood that individuals would misrepresent survey responses or that ineligible individuals would gain entry to a discussion, study remuneration was kept modest and provided for completing the screening appointment and focus group ($50 total). Oral consent procedures lasted approximately ten minutes and the screening process took approximately one hour.

#### Screening *assessments and questionnaires*

During screening, participants were interviewed about their substance use and mental health history using the Mini International Neuropsychiatric Interview and the alcohol/drug use section of the Addiction Severity Index (20, 21). A demographics questionnaire and the Brief Pain Inventory (22–24) were also used to characterize the sample.

### Initiating focus groups using videoconferencing

Following consenting and eligibility determination, focus groups were scheduled and invitations were sent via email to eligible and interested participants. Focus groups were hosted as Zoom teleconference meetings that were scheduled for up to two hours. Participants were instructed to find a space where: (1) they felt comfortable answering questions aloud regarding craving and their substance use history, (2) people could not overhear the discussion, and (3) background noise would be limited.

Two researchers joined the Zoom meeting first, which was programmed with the “Waiting room” and “Breakout room” functions enabled within the Zoom meeting platform. The ability for participants to turn on their video was disabled throughout the process. Participants could join the Zoom meeting either by computer through a link, by phone through the Zoom app, or by phone using the dial-in phone number.

**Figure 1:**
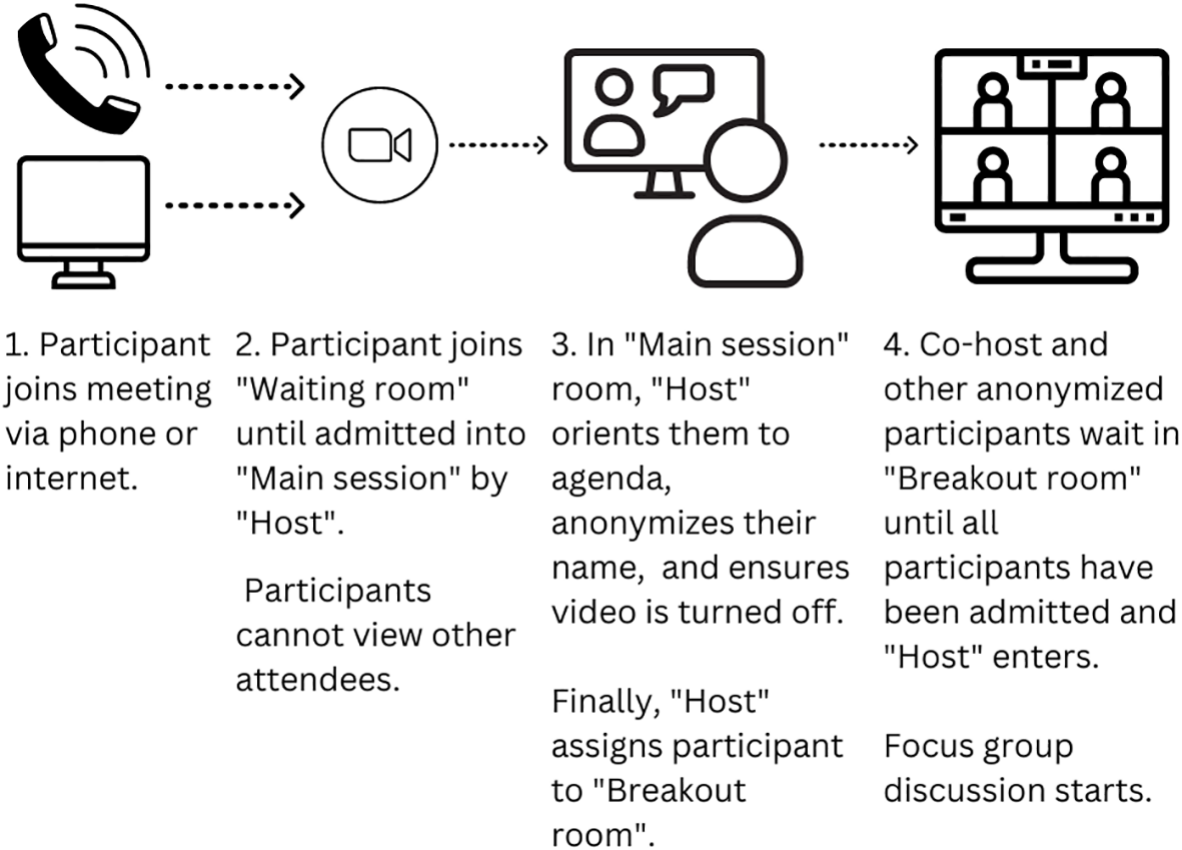
Diagram for participant flow during the virtual focus group set-up.

Upon initiating the Zoom meeting link, all participants waited in the Zoom “Waiting room,” without the ability to see other participants, before being admitted to the “Main session” room by a research team member – the “Host.” The Host then deidentified the participant, working with them to develop an anonymized name that contained a combination of first and last name initials so the participant could easily recognize themselves during the group discussion. Once ready, the Host then moved the anonymized participant into a Zoom “Breakout room” that served as the location for the focus group discussion. The Host continued the admission process with all participants until all individuals were in the same “Breakout room.” Another research coordinator – the Co-host – was also present in the “Breakout room” to greet participants and answer early questions while the group waited for the Host to admit all participants (**Figure 1**). Once all participants had been deidentified and accumulated in the “Breakout room,” the Host also joined the “Breakout room” to initiate the focus group. This set-up process took approximately 10 minutes.

### Conduct of focus groups

Either the Host or Co-host began by reading a script which described the goals of the focus group and established ground rules (e.g., avoid sharing names, do not interrupt other participants when they are speaking; see **Appendix 1**). After answering any remaining questions from attendees, the Host/Co-Host began recording the Zoom session and shared their screen to display the focus group questions, one at a time, via a PowerPoint presentation while they also read each question aloud.

Up to thirteen open-ended discussion questions were posed during the groups. Participants could look at the posed questions through the share screen function of Zoom; the Host/Co-Host also read the full question aloud so that individuals who had dialed-in to the focus group could hear the questions. Questions were discussed until they reached a point of saturation, consistent with conventional in-person focus group methodology. Both hosts were trained to probe for more information when indicated and provided a summary of discussions for each question posed. Following the summary of the discussion, participants were given the opportunity to add additional information or clarify points that might have been missed.

Immediately after the focus group ended, Hosts sent a hyperlink for a brief, optional follow-up survey that contained both open and three close-ended questions related to the acceptability of the focus group. The three close-ended questions were answered on a scale from 0 (not at all) to 10 (extremely) and included: “How much did you enjoy the focus group?”, “How comfortable did you feel during the focus group?”, and “How honest were you during the focus group?”. The open-ended question was: “What feedback do you have based on your experience in the focus group?”.

Hosts contacted participants following the focus group and arranged participant compensation payment. Options included online gift cards, mailed commercial prepaid payment cards, or direct deposit through a third-party vendor portal.

### Budget needs

This study used a Pro Zoom account in order to accommodate virtual focus groups longer than 40 minutes. Participants were compensated $50 for their participation.

### Data analysis

This report includes data on the feasibility and acceptability of virtual focus groups and whether discussion contributions differed across demographics (e.g., non-Hispanic white versus other, male versus female, history of injecting drugs versus not, interested in starting treatment/seeking treatment versus in treatment).

Descriptive statistics were used to characterize recruitment success and the demographics of the recruited sample. Discussion contributions (i.e., number of times speaking and number of words spoken) were computed for each participant based on focus group transcripts. Discussion contributions were compared across gender, race, and unique SUD subgroups using independent t-tests. Close-ended acceptability questions captured in the follow-up survey were reported using descriptive statistics. Differences in acceptability across gender and race were tested using independent t-tests.

The open-ended responses related to the acceptability of the focus group in the follow-up survey were reviewed by independent coders (CB and PS) and labeled as ‘over all favorable’ or ‘provided feedback for improvement.’ Themes for recommendations were then identified and labeled by coders. The two coders then met and compared categories and incongruencies between the qualitative data coding. If qualitative data were differentially coded, they were reviewed and discussed by coders until agreement was reached.

Descriptive statistics were used to characterize the qualitative results. Frequency of describing the focus groups as overall favorable was compared across gender and race.

## Results

### Recruitment

From February 2021 to July 2022, 2,446 individuals completed the focus group pre-screen, of which 692 (28.3%) were initially eligible. In total, 129 (18.6% of eligible) participants provided informed consent and completed screening procedures. Eligible participants who provided informed consent and ultimately participated in a virtual focus group session (n = 88, 68% of screened sample) were comparable in demographic and drug use history characteristics to those who did not participate (**Table 1**). Of the 41 participants who did not complete a focus group, 15 were ineligible because they were not in treatment nor seeking treatment and 26 did not attend the focus group and were lost contacts. Completers were mostly white (70%) and male (51%). Most participants had public health insurance, a history of injecting drugs, and one or more psychiatric comorbidities. Approximately one-third of participants reported experiencing chronic pain. Among the completers, 81% were in OUD focus groups and 19% were in StUD focus groups.

**Table 1:** Demographics and health characteristics of individuals who completed a screening session and who completed a virtual focus group session.

Demographic/Drug Use Characteristic	Screened (n = 129)	Completers (n = 88)
Age, mean ± SD years	37.9 ± 8.9	37.9 ± 9.2
% (n) Male	52 (67)	51 (45)
% (n) with yearly income ≤ $30,000	41 (53)	38 (33)
% (n) employed (part time or full time)	50 (64)	49 (43)
Race and Ethnicity		
% (n) White, not Hispanic or Latino	68 (88)	70 (62)
% (n) White, Hispanic or Latino	9 (12)	8 (7)
% (n) Black/African American	9 (12)	10 (9)
% (n) Asian	4 (5)	5 (4)
% (n) American Native	2 (3)	2 (2)
% (n) More than 1 race	7 (9)	5 (4)
Health and Substance Use Characteristics		
% (n) with public insurance	77 (99)	75 (66)
% (n) ever inject drug	59 (76)	58 (51)
% (n) with chronic pain	33 (42)	35 (31)
Past 30-day use		
Heroin, mean ± SD days	10.5 ± 12.8	10.0 ± 12.3
Cocaine, mean ± SD days	2.1 ± 5.0	2.2 ± 4.9
Amphetamines, mean ± SD days	9.1 ± 12.0	8.1 ± 11.9
% (n) in treatment	34 (44)	35 (31)
% (n) with psychiatric comorbidity	87 (112)	86 (76)

Screened and completed participants are not statistically compared and are listed to demonstrate comparable demographic and substance use history characteristics. SD, standard deviation.

**Figure 2:**
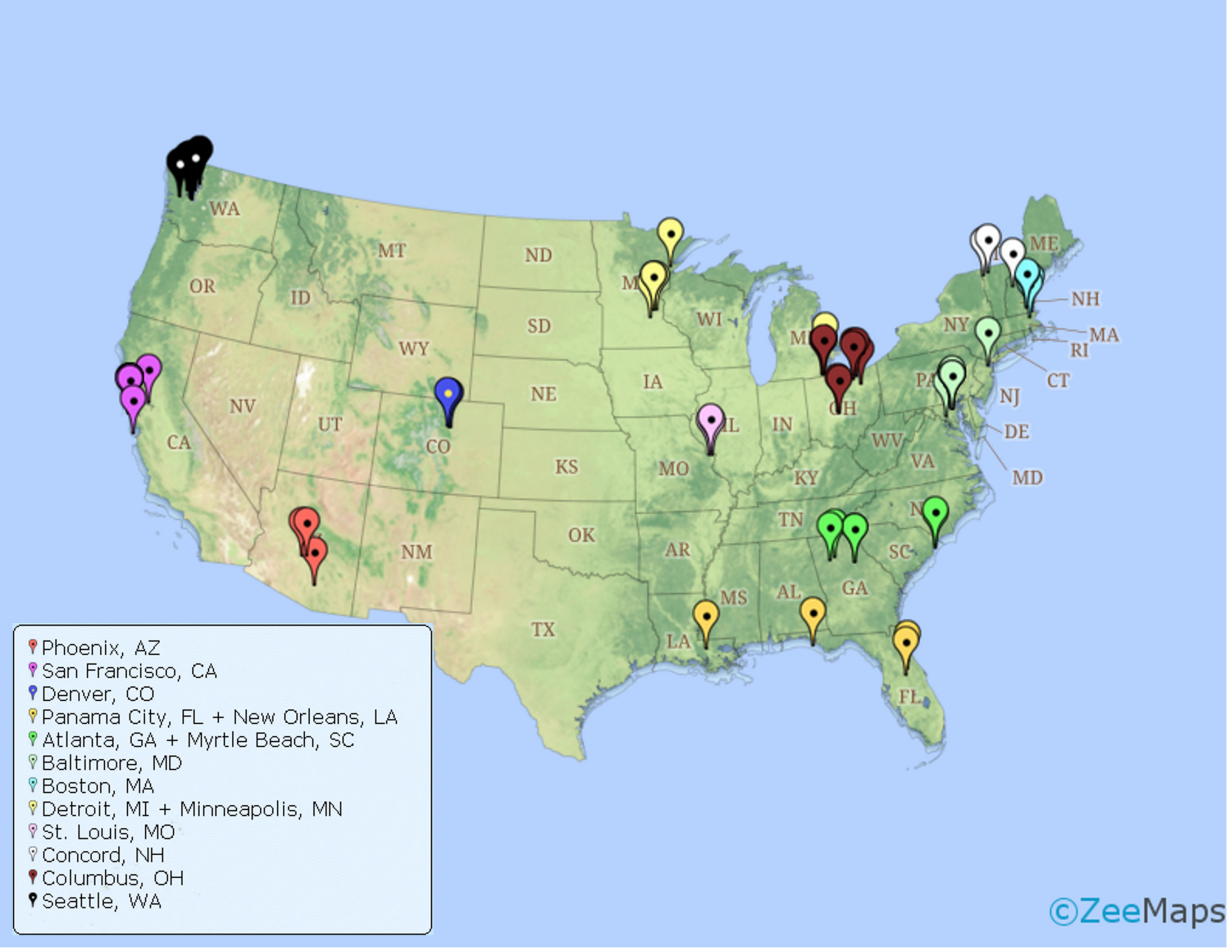
Map with pinpoints of zip codes corresponding to participants who participated in area specific focus groups (organized by color).

Twenty-six virtual focus groups were completed and participants were recruited from the general geographical areas of Atlanta, GA (*N* = 2 groups), Baltimore, MD (*N* = 4 groups), Boston, MA (*N* = 1 group), Columbus, OH (*N* = 3 groups), Concord, NH (*N* = 1 group), Denver, CO (*N* = 1 group), Detroit, MI/Minneapolis, MN (*N* = 2 groups), New Orleans, LA/Panama City, FL (*N* = 1 group), Phoenix, AZ (*N* = 3 groups), Seattle, WA (*N* = 2 groups), San Francisco, CA (*N* = 5 groups), and St. Louis, MO (*N* = 1 group, **Figure 2**). On average, focus groups consisted of 3.4 participants (*SD* = 1.0, range: 2-6).

### Participation

Discussions lasted 54.9 minutes on average (*SD* = 21.5, range: 15–132 minutes). Almost all participants attended the meeting by accessing the Zoom app (85%) and the remaining accessed the meeting by using the dial-in number associated with the Zoom invitation. Fewer than 5% of participants experienced problems related to accessing the Zoom meeting; of the 88 participants, one participant lost connection 30 minutes into their focus group and one participant experienced audio issues throughout their focus group.

### Participation for demographic groups and by substance use history

On average, participants spoke 50 times each (*SD* = 36.5, range: 1-174) and each communicated an average of 1,531 words (*SD* = 1,049, range: 5-4,412) by the time topic saturation was achieved. Of note, only 5 participants spoke fewer than 100 words. Female participants spoke significantly more times relative to male participants, (*t*(84)=-2.5, *p*=.02, *d*=0.5), but female and male participants did not significantly differ on number of words spoken, (*t*(84) = -0.8, *p* >.05) (**Tables 2**, **3**). No significant differences were detected in the number of words nor the number of times spoken among individuals who were non-Hispanic white and those that were not (number of words: *t*(84)=0.5, *p*>.05, number of times spoken: *t*(84)=0.93, *p*>.05). Additionally, participation in discussions did not differ as a function of having a history of injecting drugs nor as a function of treatment status (**Tables 2**, **3**).

**Table 2:** Mean (*SD*) number of words spoken during focus group participation as a function of gender, history of injecting drugs, and treatment status.

Demographic/Drug Use Characteristic		Number of words		p-value
		M	SD	
Gender	Male	1470.2	903.6	.46
	Female	1644.9	1223.7	
History of Injecting Drugs	Yes	1577.1	1100.8	.86
	No	1533.2	1079.0	
Treatment Status	In treatment	1451.3	1022.5	.51
	Seeking treatment	1619.4	1123.5	

**Table 3:** Mean (*SD*) number of times spoken during focus group participation as a function of gender, history of injecting drugs, and treatment status.

Demographic/Drug Use Characteristic		Number of times spoken		p-value
		M	SD	
Gender	Male	41.9	29.4	.02
	Female	62.8	47.2	
History of Injecting Drugs	Yes	42.0	30.4	.08
	No	57.6	44.4	
Treatment Status	In treatment	55.9	45.5	.45
	Seeking treatment	49.0	36.4	

### Follow-up survey results

Fifty individuals (57% of focus group participants) completed the anonymous follow-up survey after participating in the focus group. On average, participants indicated high enjoyment (*M*=8.4, *SD*=1.5, range: 4-10), comfort (*M*=9.1, *SD*=1.4, range: 2-10), and honesty (*M*=9.9, *SD*=0.3, range: 9-10) during their focus group. Ratings did not differ as a function of gender. Ratings did not differ as a function of gender or race. However, the follow-up completion rate for participants who were non-white was small (31%) compared to the rate for Whites who were not Hispanic/Latino (68%), making it difficult to draw any firm conclusions about whether experiences differed as a function of race.

Forty individuals (80%) provided responses to the qualitative feedback question “What feedback do you have based on your experience in the focus group?” Most feedback was coded as positive (73%). Eleven participants (28%) provided a response that included corrective feedback. Feedback included insufficient participant payment (*n* = 1) or undesirable payment method (*n* = 2), confusion related to not seeing faces and not knowing who was talking (*n* = 2), the need for weekend focus group sessions (*n* = 1), delays related to technical difficulties (*n* = 1), a desire to have a Moderator with lived experience (*n* = 1), more context needed for a focus group question about discussing a craving assessment (*n* = 2), and the need for an icebreaker to relieve anxiety before talking about substance use (*n* = 1).

## Discussion

Conducting remote research is a potential method for reducing barriers to research participation and increasing representation of persons that are underserved and stigmatized into research protocols. We conducted virtual focus groups with persons using opioids and/or stimulants in areas across the United States and found the method feasible and acceptable. Participant contributions did not vary as a function of demographic or drug use characteristics. We observed equitable discussion contributions across genders and that discussion contributions did not differ as a function of treatment status nor having a history of injecting drugs, two samples that represent populations with unique treatment needs and who may be facing different challenges. Finally, feedback on virtual focus groups was largely positive. Among participants who completed the follow-up survey, ratings of comfort, ability to be honest, and enjoyment were all greater than 8 on a scale from 0-10, where 10 reflected the highest levels of comfort, honesty, and enjoyment.

We recruited and retained a sample that was balanced on gender and approximated the racial composition of the U.S. (2021). Demographic and substance use history was comparable among the screened and enrolled samples, suggesting attrition was not disproportionately impacted by gender, race, nor socioeconomic status. However, the majority of the sample of participants was white. The proportion of male to female participants were comparable (51% and 49%). Notably, it was not possible to draw firm conclusions about how unique racial and ethnic groups differed in virtual focus group participation and acceptability scores. A study with targeted recruitment with oversampling of racial and ethnic subgroups of interest could help to describe any variations in discussion contributions and acceptability related to race and ethnicity. This targeted recruitment could be done by adapting and customizing advertisements, which we did not do for this research study.

A limitation of this study is the reliance on self-report data. We conducted in-depth interview assessments prior to inclusion, to assess for reporting inconsistency of medical and substance use history. Future studies could consider remote biospecimen collection to confirm opioid or stimulant use (25, 26), or obtaining a release of medical information to confirm treatment status to address this limitation.

Our focus groups were, on average, smaller than typical focus groups (27). Qualitative feedback from two individuals did indicate that there was some confusion related to not knowing who was talking because of the inability to see faces. Presumably, this issue would be compounded if more individuals participated in a virtual, anonymous, and non-visual focus group. Therefore, smaller focus groups may be more appropriate in virtual formats when participants are anonymized. As an alternative option, it may be possible to conduct moderate sized focus groups in which all participants agree to allow video capability.

Notably, qualitative feedback indicated that some participants (n = 2) had difficulty understanding group questions related to the target topics. Providing visual aids to supplement focus group discussion prompts could eliminate confusion. Visual aids could be sent by email or text message and could be provided prior to the discussion group or at the screening session.

We did not collect data on whether participants had previous experience participating in research studies so we do not know whether they differ in this regard from samples of participants recruited for in-person studies. Because of the primary purpose of this study, we excluded individuals who are pregnant from participating in the research study, which is a limitation in the generalizability of these findings. However, virtual focus groups are likely a promising research method for this highly stigmatized group and should be further explored and utilized.

Importantly, most participants provided positive feedback, suggesting that any alterations to the current procedures are minimal. However, 20% of the eligible sample did not complete the focus group, suggesting some barriers prevented them from joining the focus group appointment. Scheduling an event where multiple people can attend is difficult and study attrition is a universal issue in research. However, to increase flexibility and retention, one-on-one interviews could be considered to gather patient-centered feedback. It is possible that this method could encourage discussion for some participants who do not feel comfortable disclosing use history in a group. Alternatively, some find focus group settings an effective way to share, especially among peers with shared lived experiences.

In the future, tailoring approaches to individual preferences should be considered (i.e., offering both one-on-one interviews and focus group discussions). Further, there is opportunity to expand this method to other geocultural locations, depending on reliable access to internet. Such applications should ensure moderators/hosts have cross-cultural sensitivity, knowledge of cultural norms and communication styles, and understanding of language nuances.

Virtual focus groups may provide a valuable method for collecting patient-centered qualitative feedback, which is an important tool in the development and refinement of novel interventions. In addition, such groups could be useful in other, related areas, such as in the assessment of existing treatments, and in policy decisions. With respect to the development of new tools and interventions, collection of patient-centered feedback is a requirement for receiving FDA approval for a qualified patient-reported clinical outcome assessment (28, 29). These methods and our preliminary acceptability and feasibility data offer a strategy to collect patient-centered feedback in a virtual platform from a unique and at times hard to reach population.

## Data availability statement

The raw data supporting the conclusions of this article will be made available by the authors, without undue reservation.

## Ethics statement

The studies involving humans were approved by The Johns Hopkins Medicine IRB. The studies were conducted in accordance with the local legislation and institutional requirements. The ethics committee/institutional review board waived the requirement of written informed consent for participation from the participants or the participants’ legal guardians/next of kin because oral consent procedure was followed with approval from the Johns Hopkins Medicine IRB.

## Author contributions

CB: Conceptualization, Data curation, Formal Analysis, Funding acquisition, Investigation, Methodology, Project administration, Resources, Supervision, Visualization, Writing – original draft, Writing – review & editing. BP: Data curation, Project administration, Supervision, Visualization, Writing – review & editing. PS: Data curation, Formal Analysis, Writing – review & editing. KD: Writing – original draft. RD: Conceptualization, Funding acquisition, Methodology, Supervision, Writing – review & editing. ES: Conceptualization, Investigation, Methodology, Supervision, Visualization, Writing – review & editing.

## Appendix 1. Focus Group Introduction Script

Thank you for joining us for this focus group discussion. We are researchers at the Johns Hopkins University School of Medicine, and we are interested in learning more about ways to help individuals who have a history of opioid/stimulant use related problems. We especially want to know and understand craving, and the best ways to relieve craving. My name is _________________ and I will be facilitating the discussion today and asking questions for the group to discuss.

First - We can’t thank you enough for your time and effort! As a reminder this is completely voluntary, if at any point you no longer wish to participate, please let us know.

Today we are doing an anonymous focus group discussion, we have hidden everybody’s name and videos. Please avoid identifying yourself during this discussion. We want everyone to feel comfortable.

As a reminder, we anticipate this discussion will take approximately one to two hours (depending on the size of the group). We will also ask you to complete a brief follow-up survey to let us know if you have any feedback for our group.

For compensation, we will send you a $50 e-voucher for Amazon, a $50 commercial bank gift card, or $50 direct deposit or check. We will follow up separately to make sure we deliver your payment to the appropriate email address and recipient.

During the discussion, there may be topics that do not apply to you or that you do not feel comfortable answering. That is completely okay. We just ask that you share whatever you are comfortable sharing. In addition to understanding individual experiences, we also are interested in knowing what are common experiences. Therefore, if you simply agree with what somebody says, we encourage you to let us know.

During the discussion, please be respectful of everyone’s’ opinions and do not interrupt somebody when they are talking. If your experiences are different from other participants’, please let us know but please do so in a respectful way.

If at any time, you need to step away, please let us know. When you have returned, please announce that you’ve returned.

Does anybody have any questions before we get started?
